# Superparamagnetic Oxygen-Loaded Nanobubbles to Enhance Tumor Oxygenation During Hyperthermia

**DOI:** 10.3389/fphar.2019.01001

**Published:** 2019-09-11

**Authors:** Sara Zullino, Monica Argenziano, Shoeb Ansari, Roberta Ciprian, Lucia Nasi, Franca Albertini, Roberta Cavalli, Caterina Guiot

**Affiliations:** ^1^Molecular Imaging Center, Department of Molecular Biotechnology and Health Sciences, University of Torino, Torino, Italy; ^2^Department of Neuroscience, University of Torino, Torino, Italy; ^3^Department of Drug Science and Technology, University of Torino, Torino, Italy; ^4^Consiglio Nazionale delle Ricerche, Istituto dei Materiali per l’Elettronica ed il Magnetismo, Parma, Italy

**Keywords:** theranostics, ultrasound, nanobubbles, SPIONs, oxygen, tumor, magnetic hyperthermia

## Abstract

Tumor oxygenation is a critical issue for enhancing radiotherapy (RT) effectiveness. Alternating RT with hyperthermia improves tumor radiosensitivity by inducing a massive vasodilation of the neoangiogenic vasculature provided the whole tumor is properly heated. The aim of this work was to develop superparamagnetic oxygen-loaded nanobubbles (MOLNBs) as innovative theranostic hyperthermic agents to potentiate tumor oxygenation by direct intracellular oxygen administration. Magnetic oxygen-loaded nanobubbles were obtained by functionalizing dextran-shelled and perfluoropentane-cored nanobubbles with superparamagnetic iron oxide nanoparticles. Magnetic oxygen-loaded nanobubbles with sizes of about 380 nm were manufactured, and they were able to store oxygen and *in vitro* release it with prolonged kinetics. *In vitro* investigation showed that MOLNBs can increase tissue temperature when exposed to radiofrequency magnetic fields. Moreover, they are easily internalized by tumor cells, herein releasing oxygen with a sustained kinetics. In conclusion, MOLNBs can be considered a multimodal theranostic platform since, beyond their nature of contrast agent for magnetic resonance imaging due to magnetic characteristics, they showed echogenic properties and can be visualized using medical ultrasound.

## Introduction

Oncological hyperthermia (HT) is a therapy consisting in increasing tumor temperature between 40°C and 45°C for about 1 hour in two to three weekly sessions often concomitantly with radiotherapy (RT) and sometimes radiochemotherapy (Falk and Issels, 2001; Hildebrandt et al., 2002; van der Zee, 2002;Wust et al., 2002).

Effectiveness is critically related to the ability of heating the tumor almost uniformly and persistently during the HT session. As a matter of fact, the reaching of a breakpoint temperature, conventionally assumed cumulatively equivalent to 43°C according to the definition of cumulative equivalent minutes at 43 °C (Sapareto and Dewey, 1984) for a significant fraction and over a significant volume of the tumor (90%), is mandatory for clinical success in most of the tumors (Dewhirst and Sim, 1984; Thrall et al., 2000).

External heating devices based on microwaves, radiofrequency (RF), ultrasound (US), or infrared are often inadequate for treating the whole tumor volume, especially deep-seated ones (Wust et al., 2002), and although the physiological vasodilatory-based temperature control can be in principle counteracted, it would require multisite direct invasive temperature monitoring, which is uncomfortable, harmful, and possibly unethical.

Invasive temperature monitoring shows an extreme inhomogeneous distribution and a typical initial delay both for temperature rise and hyperemia (Kim et al., 1976; Gerweck, 1977; Gerweck et al., 1979; Guiot et al., 1998).

The need for endogenous heat generation mechanisms led to the investigation of the so-called magnetic fluid hyperthermia (MFH), consisting in the *in situ* administration of a stable colloidal suspension of biocompatible superparamagnetic iron oxide nanoparticles (SPIONs), which can be activated by external magnetic fields (Hildebrandt et al., 2002). The physical bases and the possibility of tailoring treatments based on the fine tuning of a few parameters are well described in the literature (Spirou et al., 2018).

Clinical studies of MFH based on Food and Drug Administration–approved and marketed systems (MagForce^®^ activated by MFH^®^ 300F or NanoActivator^®^) in combination with RT, even in very challenging tumors such as recurrent glioblastoma (Maier-Hauff et al., 2007) and metastatic bone tumors (Matsumine et al., 2011), showed very promising results.

A number of research are still open to innovative approaches able to improve the biological benefits of the MFH (Ansari et al., 2019).

Magnetic nanobubbles (NBs) containing paclitaxel and decorated with herceptin and ultrasmall superparamagnetic iron oxide have been studied for targeted drug delivery and multimodal imaging in breast cancer cells (Song et al., 2017). Recently, pemetrexed- and pazopanib-carrying NBs with magnetic responsiveness and US sensitivity properties have been designed as new theranostic system for targeted non–small cell lung cancer (Şanlıer et al., 2019).

The rationale for the combination of HT and RT is based on some “complementary” effects, namely, the fact that tumor cells are more radioresistant when their microenvironment is hypoxic (Sapareto and Dewey, 1984) and acidic (Dewhirst and Sim, 1984) and, during the S phase, when the cell is more sensitive to heat (Thrall et al., 2000).

Other indirect effects are even more important. Due to the abnormal structure of the angiogenically driven tumor vasculature, which is irregular and leaky, heat is less effectively dissipated and can both damage the microvasculature and induce local HT (Hildebrandt et al., 2002). Therefore, improved blood circulation reduces hypoxia and increases environmental pH transitorily reversing radioresistance (and chemoresistance).

Being tumor oxygenation one of the main targets of the hyperthermic treatment, here we investigated the use of oxygen nanocarriers “decorated” with SPIONs, hereinafter named magnetic oxygen-loaded nanobubbles (MOLNBs), which combined the magnetic-based increase in temperature with a direct oxygenation effect in the tumor volume.

Oxygen-loaded microbubbles have been already employed both for sonography as US contrast agents and for oxygen delivery to the tumor hypoxic microenvironment (Eisenbrey et al., 2018; Yang et al., 2018).

Nanobubbles are spherical core-shell structure, filled with gas or vaporizable compounds, with sizes in the nanometer order of magnitude. They were designed to improve stability in the circulation and to allow extravasation and accumulation in tumor tissue by Enhanced permeability and retention (EPR) effect (Cavalli et al., 2016).

Previously, theranostic polymer-shelled NBs have been designed showing the capability to act as drug carriers and US imaging systems (Cavalli et al., 2015). Oxygen-loaded nanobubbles (OLNBs) were already manufactured, patented, and proposed by our research team for application to other pathologies (Cavalli et al., 2009; Magnetto et al., 2014; Banche et al., 2015; Prato et al., 2015), and preliminary studies on the coupling of SPIONs with OLNBs were proposed as well (Zullino et al., 2015).

## Materials and Methods

### Materials

Unless otherwise stated, the materials employed were purchased from Sigma-Aldrich (St. Louis, MO, USA). All the reagents were of analytical grade. Epikuron 200^®^ was a kind gift from Cargill.

### Synthesis of SPIONs

Superparamagnetic iron oxide nanoparticles (Fe_3_O_4_) were prepared tuning a protocol previously reported (Zhang et al., 2015).

Weighed amounts of FeCl_2_ 4H_2_O (0.99 g) and FeCl_3_ 6H_2_O (2.703 g) were added to deionized distilled water (50 mL) under continuous nitrogen purging and stirred at 85°C until dissolved. Then, standardized ammonium hydroxide solution (14 mL) was dropwise added, followed by stirring at 85°C for 1 h. After the system reached a precipitation state, it was allowed for cooling and settling of the precipitation in the bottom of the flask. After precipitation, the supernatant liquid is first decanted carefully without disturbing precipitation. The precipitate was washed with water, and then it was allowed to expose in air for 24 h.

The obtained SPION nanoparticles were stored at 4°C, until the MOLNB preparation. Then they were added to the preformed NB nanosuspension, exploiting the electrostatic interaction with the dextran sulfate shell.

### Preparation of MOLNB formulations

**Figure 1:**
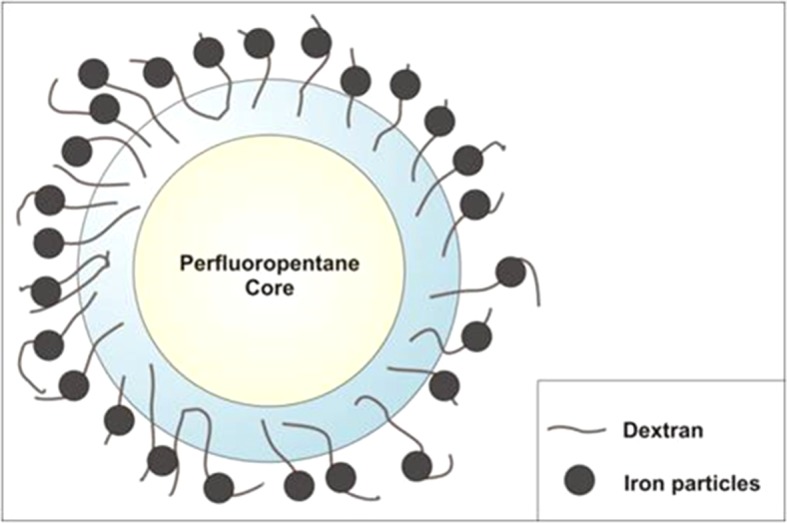
Sketch of MOLNB structure [dextran NBs covered by Fe_3_O_4_ nanoparticles (not to scale)].

An ethanolic solution of Epikuron 200^®^ (1% wt/wt) and palmitic acid as cosurfactant (1% wt/wt) was added under stirring to perfluoropentane (PFP, C_5_F_12_) at room temperature. Then, a volume of ultrapure water was slowly added to the mixture under mild stirring until the formation of an emulsion. The system was then homogenized for 3 min using a high-shear homogenizer (Ultraturrax, IKA, Germany) in an ice bath. The nanosuspension was saturated with O_2_ until reaching a 35 mg/L concentration in the aqueous medium. Then, an aqueous solution of dextran sulfate sodium salt (1% wt/wt) was added drop-wise to form the NB polymeric shell under an oxygen purge. Finally, 1 or 2 mg/mL of SPIONs was added drop-wise to the suspension under mild stirring at room temperature (**Figure 1**).

For the preparation of fluorescent NBs, 6-coumarin was loaded into the NB core by addition of the fluorochrome directly to PFP solution. The same preparation protocol used for blank formulations was employed. As control, blank dextran-shelled OLNBs were also prepared, without the addition of SPIONs.

### Physicochemical Characterization of NB Formulations

Average hydrodynamic diameter, polydispersity index, and zeta-potential values of the NB formulations and SPIONs were determined by photocorrelation spectroscopy using a laser light scattering at a temperature of 25°C and a scattering angle of 90°. The samples were diluted with filtered water (1:30 dilution). Each measured value was the average of 10 readouts. For zeta-potential determination, samples of the NB formulations were placed in the electrophoretic cell of the same instrument. The electric field was set to 14 V/cm. The viscosity of the NB formulation was determined at 25°C using a Ubbelohde capillary viscosimeter (Schott Gerate, Mainz, Germany). The osmolarity was determined at 25°C using a Knauer osmometer.

The morphology of blank OLNB, MOLNB, and SPIONs was observed by transmission electron microscopy (TEM) in conventional and high-resolution (HR) modes, by using a JEOL2200FS microscope working at 200 keV. The diluted NB aqueous suspensions were sprayed on Formwar-coated copper grid and air dried before observation.

### Determination of NB Physical Stability Over Time

The physical stability of blank OLNB and MOLNB was evaluated over time. The average diameter and morphology of the NB formulations stored at 4°C were determined up to 2 months.

### Ultrasound Imaging Evaluation

**Figure 2:**
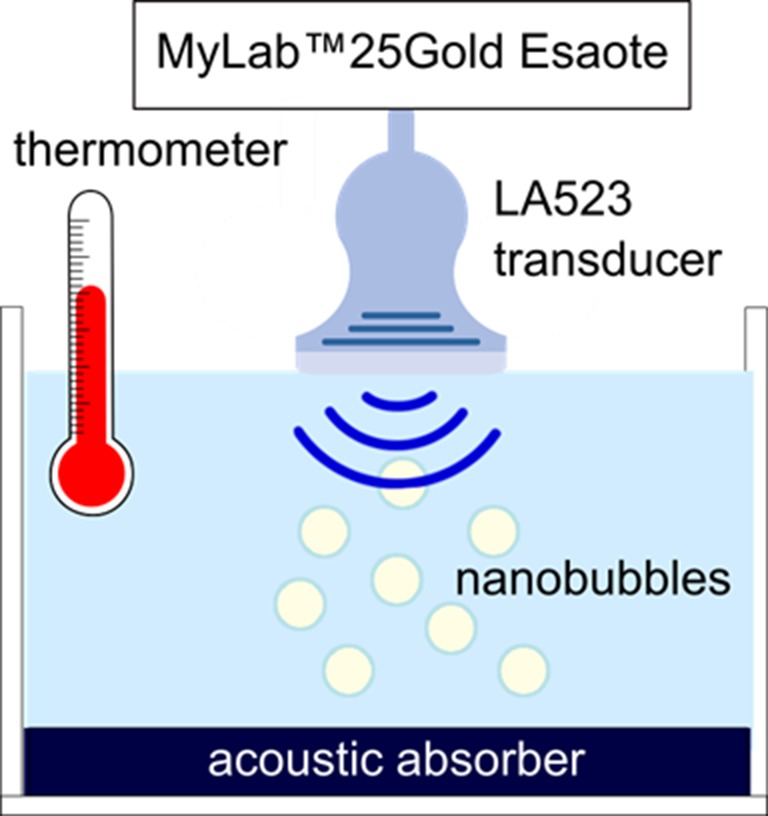
Experimental setup used for US imaging.

B-mode US imaging was carried out to investigate the echogenicity of the OLNBs and the MOLNBs. Nanobubble aqueous suspension at concentration ∼1 × 10^10^ NBs/mL was pipetted in a tank containing demineralized water and gently mixed using a magnetic stirrer in order to have a homogeneous suspension. At the bottom of the tank, an acoustic absorbing pad was placed to minimize acoustic reflections. The experiment was performed at increasing temperatures: 25°C, 37°C, and 41°C with the aim to mimic the room temperature and the physiological and hyperthermic conditions. Nanobubbles were insonified by a US clinical echomachine (MyLab™ 25Gold; Esaote, Genova, Italy), equipped with a linear array transducer (LA523, 7.5 MHz central frequency; Esaote) operating in B-mode using the small parts imaging preset. A sketch of the experimental setup is shown in **Figure 2**. B-mode cineloops were acquired at increasing Mechanical Index (MI), i.e., increasing acoustic power, maintaining the same imaging preset. B-mode cineloops of demineralized water in the absence of NBs were also acquired as reference.

### Hyperthermic and Magnetic Measurements

Hyperthermia properties were evaluated by calorimetric measurements by means of an AC commercial applicator (nanoScale Biomagnetics DM100, Zaragoza, Spain).

Magnetic oxygen-loaded nanobubbles decorated with SPIONs at a concentration of 1 or 2 mg/mL were exposed for 10 min to an RF alternating magnetic field of amplitude = 250 Oe, frequency = 429 kHz. The temperature increase was measured with an optical fiber thermometer to avoid the coupling with the RF field.

Isothermal magnetization curves at room temperature were measured by an alternating gradient force magnetometer.

### *In Vitro* Oxygen Release Studies

*In vitro* oxygen release from MOLNBs and in the absence of SPIONs was investigated using the dialysis bag technique. The donor phase, consisting of 3 mL of MOLNBs or OLNBs, was placed in a dialysis bag (cellulose membrane, 12 kDa) Then, it was put in 45 mL of saline solution (NaCl 0.9% wt/vol) whose oxygen concentration was previously reduced (up to 1 mg/L) with a N_2_ purge in order to mimic hypoxic conditions. The concentration of oxygen released by diffusion from MOLNBs into the receiving phase was monitored for 24 h, using an oximeter (HQ40d model; Hach).

The *in vitro* oxygen release kinetics was evaluated at different temperatures (i.e., 25°C, 37°C, and 41°C) to simulate ambient and normal body temperatures and hyperthermic sessions, respectively.

Oxygen release measurements under the effect of the magnetic field were prevented by the metallic nature of the oximetry probe and its interference with the AC applicator.

### Cell Internalization

TUBO cells, a cloned rat Her2/neu+ cell line established from a lobular carcinoma of a BALB-neuT mouse, were cultured in high-glucose Dulbecco’s modified eagle medium (DMEM), containing 20% fetal bovine serum, 2 mM l-glutamine and 1% penicillin-streptomycin in a humidified CO_2_/air incubator at 37°C. For NB uptake evaluation, TUBO cells were plated in 6-well plates on glass coverslips and incubated in DMEM medium for 24 h with/without 10% vol/vol 6-coumarin–labeled NBs in a humidified CO_2_/air incubator at 37°C. After 24-h incubation with 6-coumarin–labeled NBs, TUBO cells were fixed, stained with 4′,6-diamidino-2-phenylindole (DAPI) to visualize cells nuclei, and then visualized by microscope. Fluorescence images were acquired by an LSM710 inverted confocal laser scanning microscope (Carl Zeiss, Oberkochen, Germany) equipped with a Plan-Neofluar 63 × 1.4 oil objective, which allowed a field view of at least five cells. Wavelength of 488 nm was used to detect fluorescent OLNBs and of 460 nm to detect the labeled nuclei. The acquisition time was 400 ms.

### Statistical Analysis

Data are expressed as means ± SD. Significance between experimental groups was determined by one-way analysis of variance followed by the Bonferroni multiple-comparisons posttest using GraphPad InStat software (San Diego, CA, USA). *p* ≤ 0.05 was considered significant.

## Results

### Physicochemical Characterization of NB Formulations

Decorated SPIONs and oxygen-loaded NBs were manufactured using biocompatible components, tuning the preparation protocol, which was previously reported (Argenziano et al., 2017).

The physicochemical parameters of OLNBs before and after the addition of SPIONs are reported in **Table 1**.

**Table 1:** Physicochemical characteristics of NB formulations.

Formulation	Average diameter ± SD (nm)	Polydispersity Index	Zeta Potential ± SD (mV)
Blank OLNBs	375.5 ± 20.4	0.21 ± 0.01	−30.4 ± 3.45
Fluorescent OLNBs	378.2 ± 26.3	0.21 ± 0.01	−28.8 ± 3.68
MOLNBs	385.4 ± 25.8	0.19 ± 0.02	−19.72 ± 1.24

The zeta-potential of SPIONs was +11.63 mV. The positive charge is suitable for obtaining a binding on the negative polymer shell by electrostatic interactions. The presence of SPIONs on the polymer shell produced a decrease of the MOLNB zeta potential of about 30%. This behavior can be related to the electrostatic binding of SPIONs and the partial shielding of the surface charge of NBs.

The pH of the NB nanosuspension was 5.86, the viscosity was about 1.42 cP, and the osmolarity was 354 mOsm, values suitable for future clinical translation. These parameters were not affected by the presence of the SPIONs. Moreover, the nanosuspension was homogeneous and stable over 2 months.

**Figure 3:**
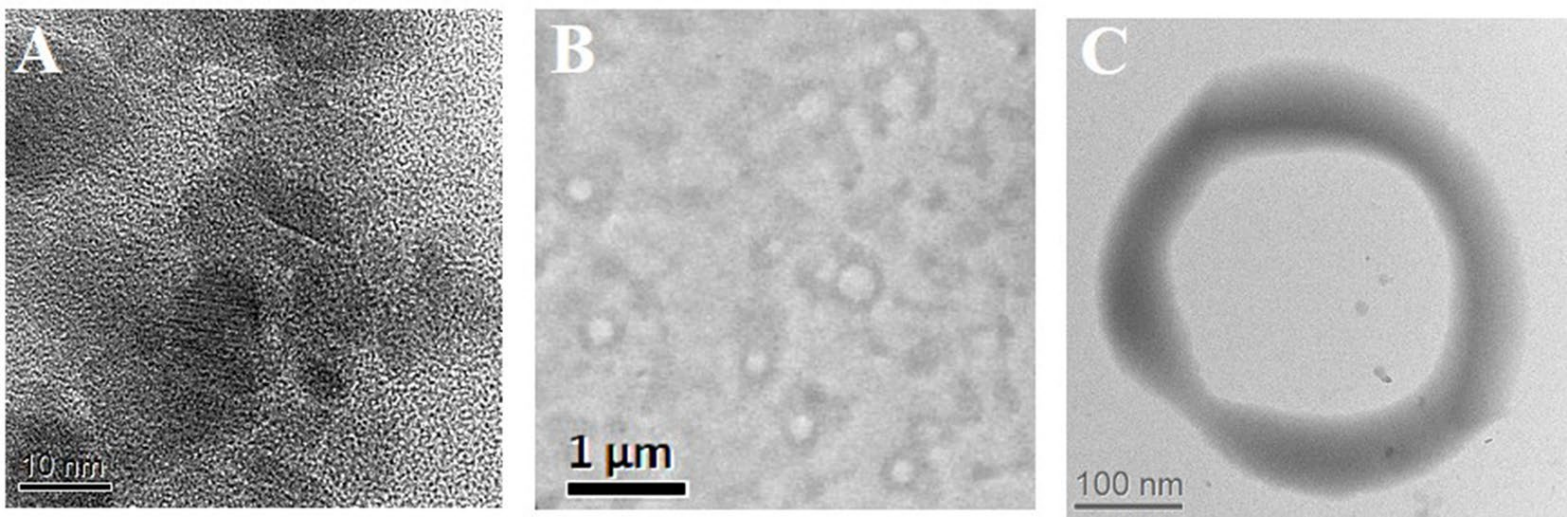
**(A)** HRTEM image of SPIONs; **(B, C)** TEM images of blank-shelled OLNBs at different magnifications.

**Figure 3** shows the TEM images of both SPIONs (**Figure 3A**) and blank dextran-shelled OLNBs (**Figures 3B, C**). The spherical and hollow morphology of the NBs is clearly visible in the images.

**Figure 4:**
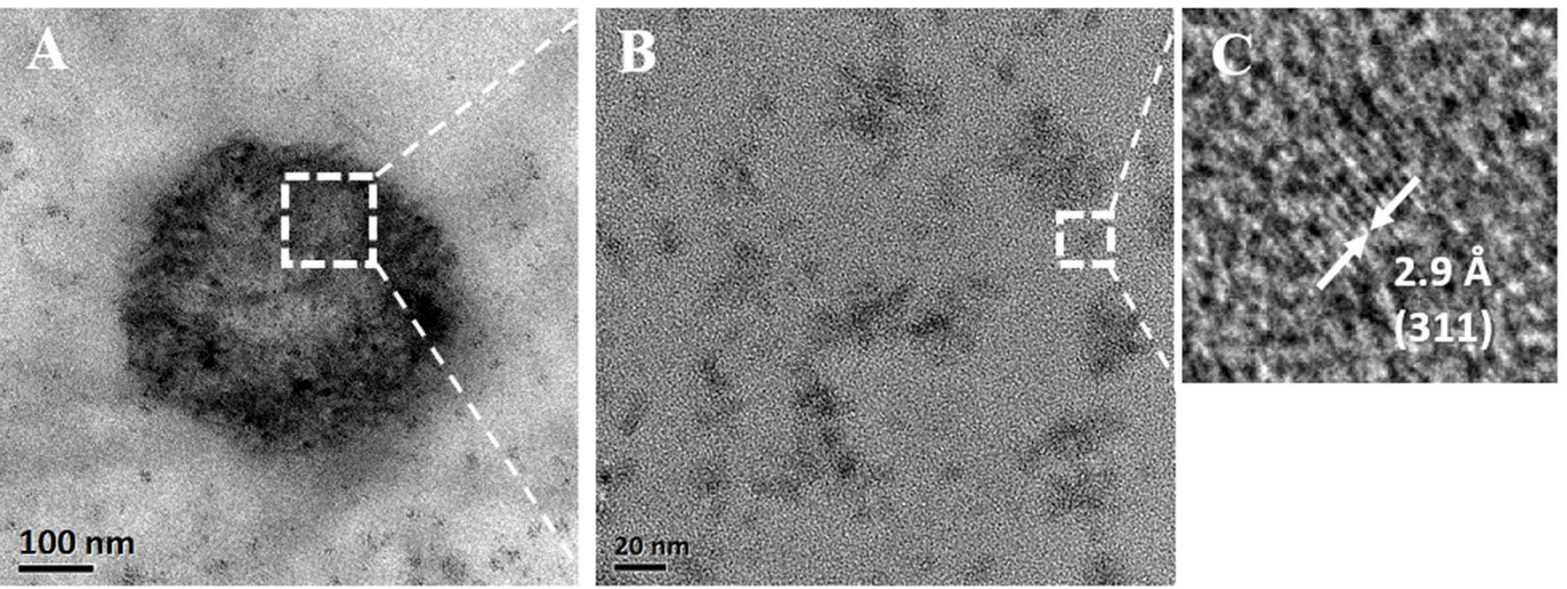
**(A)** TEM image of an MOLNB; **(B)** HRTEM image of the square region in **(A)**; **(C)** HRTEM image of the square region in **(A)** showing the (311) lattice planes of a Fe_3_O_4_ nanoparticle.

The TEM studies of functionalized nanobubbles (MOLNBs) revealed a more contrasted shell with respect to OLNBs, maintaining the classic spherical shape, as shown in **Figure 4A**. By progressively increasing the magnification, the “granular” structure of the SPIONs in the shell becomes evident (**Figures 4B, C**). An average diameter of about 390 nm was confirmed for MOLNBs.

Fluorescent OLNBs were visible under fluorescence microscope and the morphology and physicochemical properties were the same as previously described.

*In vitro* stability studies showed that the nanosuspension was homogeneous and stable over 2 months.

### Ultrasound Imaging Evaluation

**Figure 5:**
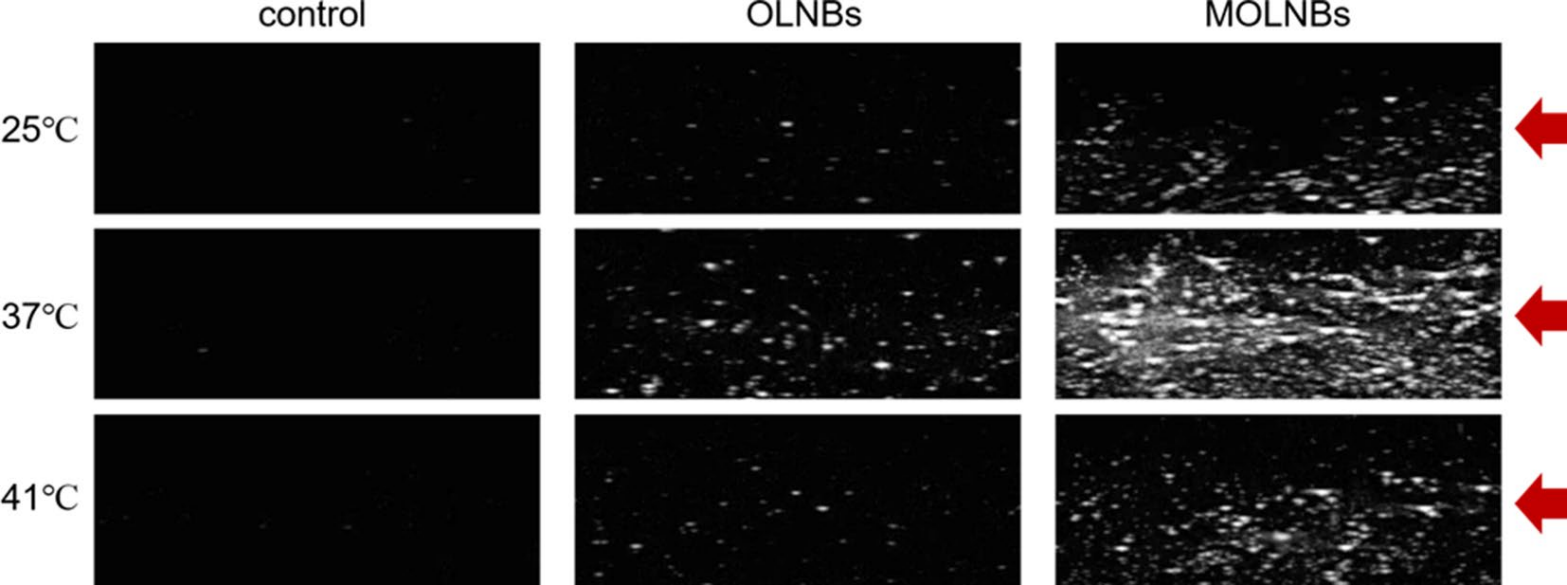
Snapshots of the B-mode cineloops of control experiment (demineralized water only), OLNBs, and MOLNBs at MI = 1.1 (i.e., maximum acoustic power) and different temperatures. Red arrows indicate the focus of the US probe.

Ultrasound experiments revealed that MOLNBs showed an increased echogenicity with respect to OLNBs (**Figure 5**). The ability of both the OLNBs and MOLNBs to generate an echogenic response was enhanced at 37°C, as visually demonstrated. The insonation affected the detectability increasing the volume size of NBs favoring the vaporization, due to acoustic droplet vaporization (ADV) phenomenon (Kripfgans et al., 2004). A decrease in the echogenicity was observed at 41°C, probably because most of the NBs showed a different vaporization behavior. We could speculate that this could be related to the phase transition of phospholipids (i.e., phosphatidylcholine) present in OLNBs from the gel to the liquid crystalline state. This effect was previously observed also for Sonovue^®^ (Bracco, Italy), a commercial US contrast agent consisting of microbubbles loaded with SF6 and coated with a phospholipidic shell (Guiot et al., 2006).

### Hyperthermic and Magnetic Measurements

**Figure 6:**
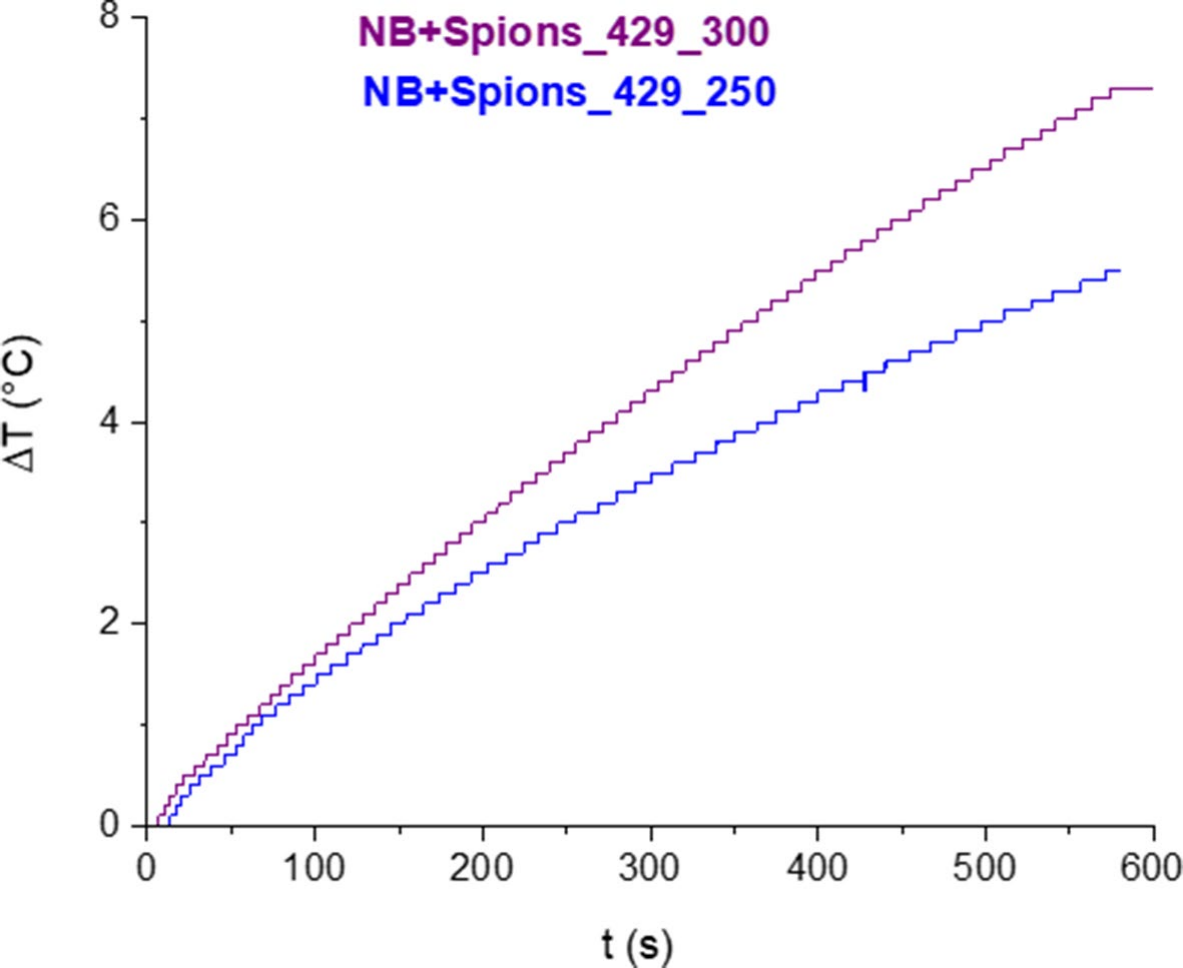
Variation of temperature as a function of time under exposure of RF magnetic field for two different AC field amplitudes.

A first simulation of the hyperthermic session was performed by exposing for 10 min a colloidal suspension of MOLNBs to an RF magnetic field. A temperature increase of the sample was observed during the RF field application. In **Figure 6**, the time dependence of the suspension temperature under irradiation is reported for two different NB concentrations. After 10 min of exposure, a temperature increase of 5°C (at field amplitude of 250 Oe) or 7°C (at field amplitude of 300 Oe) for the 2 mg/mL SPION concentration was attained.

The specific heat absorption rate (SAR), expressed in W/g, was evaluated by using the following expression (Eq. 1):

(1) $$\text{SAR}=\frac{c\cdot \rho }{C}\cdot \frac{\mathrm{dT}}{\mathrm{dt}}$$

where *c* is the specific heat capacity of the sample (for diluted solutions as in the present case, *c* is equal to the heat capacity of the solvent, assumed to be 4.18 J/g °C), ρ is the density of the solvent (equal to 1 g/mL), *C* is the concentration of the magnetic NB in the solvent (2 g/L), and *dT/dt* is the heating rate of the sample. The heating rate was calculated by fitting the temperature increase ΔT with a linear trend at early times, i.e., first 10 seconds of the experiment.

Depending on the field amplitude, the SAR values range between 37.7 and 46.1 W/g.

The deviation from the linearity at longer times of the temperature increase is dictated by the combined effects of heating power and thermal losses. The temperature increase of the solution can be attributed to the magnetic properties of MOLNBs, as confirmed by room temperature isothermal magnetization measurements.

**Figure 7:**
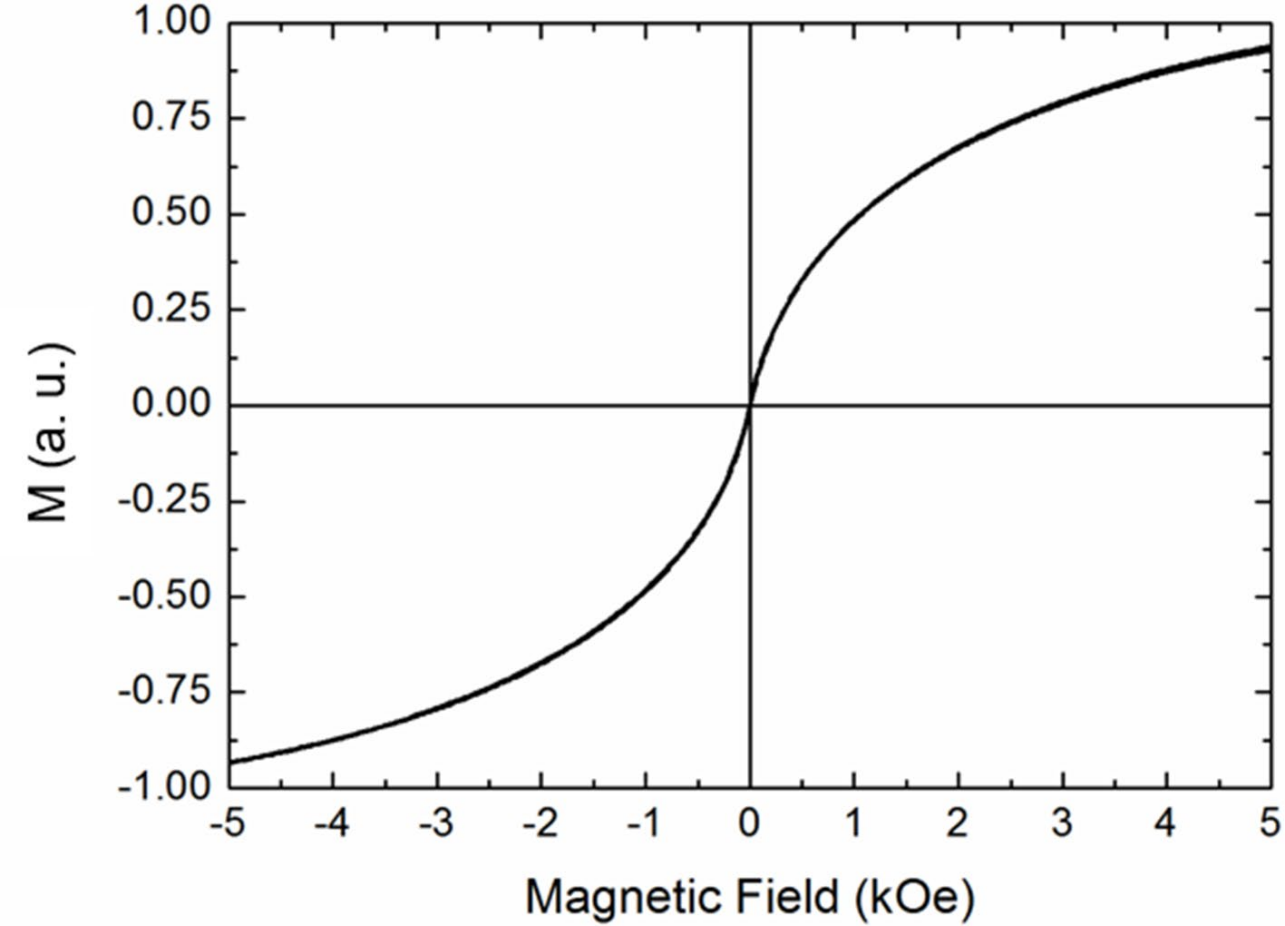
RT magnetization curve for MOLNBs.

In **Figure 7**, hysteresis loop measurements are reported, confirming the superparamagnetic behavior of magnetic NBs, characterized by no remanence and no hysteresis.

### *In Vitro* Oxygen Release

**Figure 8:**
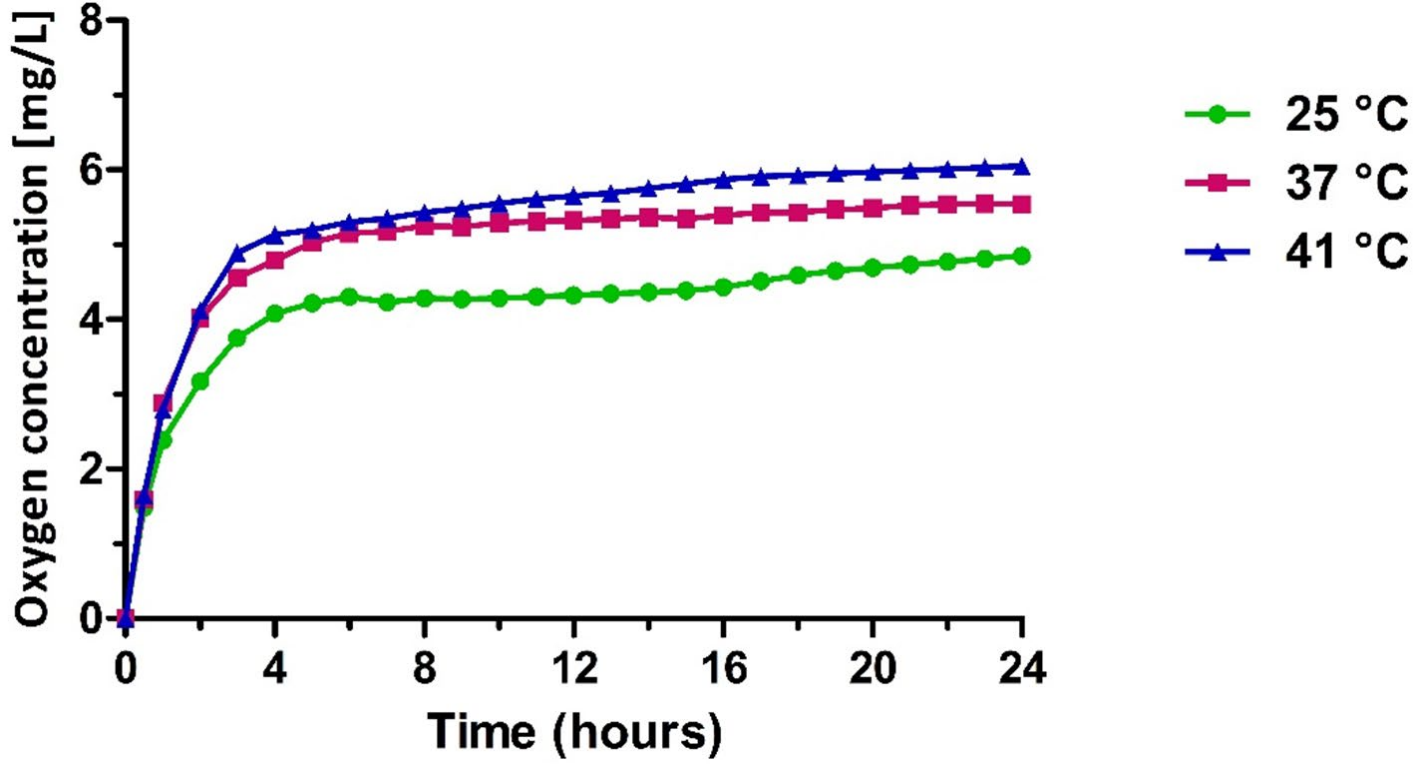
*In vitro* oxygen release from MOLNBs at different temperatures (i.e., 25°C, 37°C, and 41°C).

The release studies showed the capability of OLNBs and MOLNBs to store and release oxygen. The *in vitro* oxygen release kinetics from MOLNBs into a hypoxic receiving phase showed a biphasic profile (**Figure 8**). In the first 4 h, oxygen was rapidly released from MOLNBs, reaching an oxygen concentration between about 4 and 6 mg/L at 25°C and 41°C, respectively. Subsequently, an almost constant and slow oxygen release was obtained. After 24 h, the oxygen concentration in the external receiving phase kept constant, because an equilibrium between the internal and external phases was reached. A temperature-dependent release profile was observed. The presence of SPIONs on NB shell did not affect the oxygen release capability. Indeed, the same *in vitro* release profiles were obtained for OLNBs at all the temperatures.

### Cell Internalization

**Figure 9:**
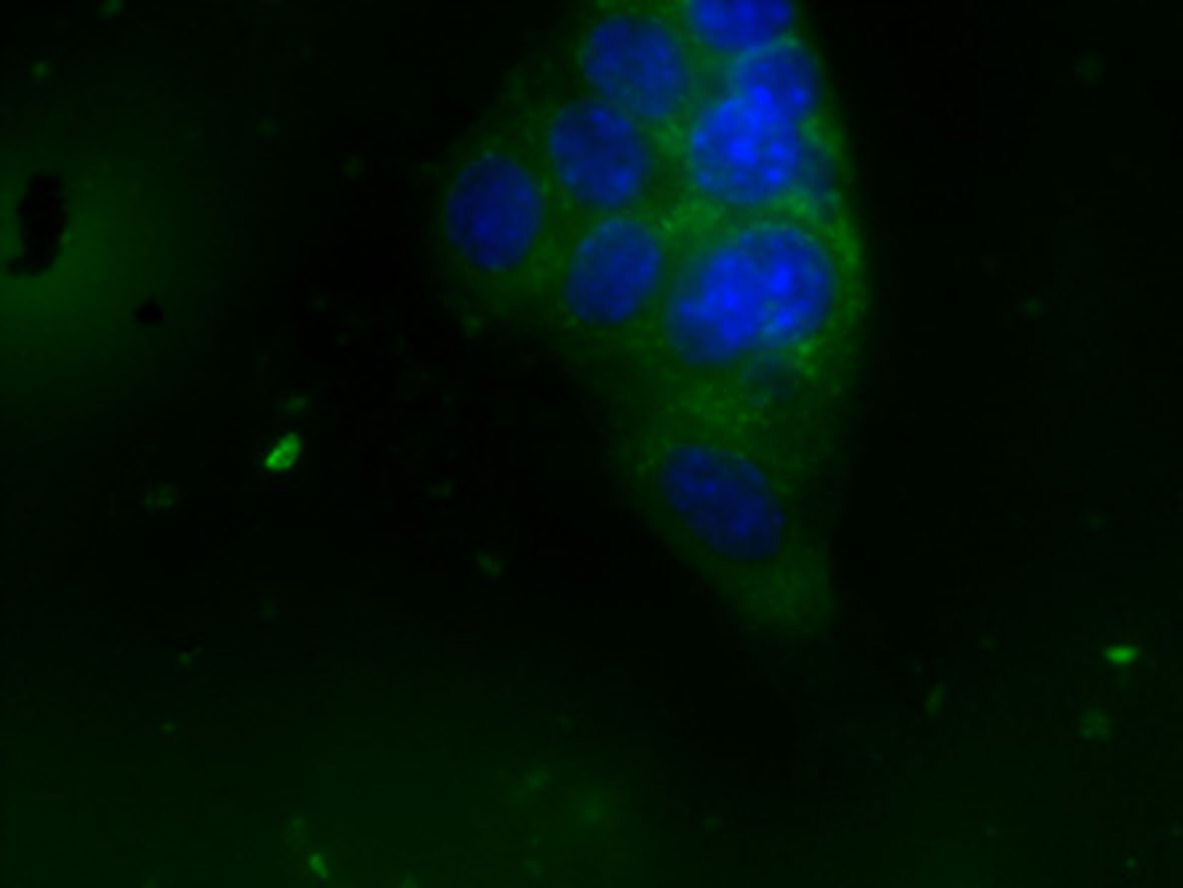
OLNB internalization by TUBO cell line. Cell nuclei after DAPI staining (in blue; OLNBs, in green). Magnification ×63.

Fluorescence microscopy was used to verify if NBs were internalized by breast cancer *in vitro* model. Results show that NBs were avidly internalized by TUBO cells and were localized only in cellular cytoplasm compartment (**Figure 9**). This result indicates that MOLNBs were able to deliver a gas or a drug directly inside the cancer cells.

## Discussion

Drug-loaded NBs have been previously studied, showing an increase in drug blood lifetime, extravasation capability, and accumulation in tumors by passive targeting, exploiting the EPR effect, in comparison with microbubble (Cavalli et al., 2016). Oxygen-loaded nanobubbles have been developed to store and deliver oxygen in hypoxic tissues. Oxygen-loaded nanobubbles are also very attractive nanocarriers for their potential application in cancer therapy, due to their theranostic property. Moreover, they could play a key role as radiosensitizer in RT, where the enhancing effect of the oxygen is well known. In the literature, magnetic oxygen-loaded microbubbles were previously described for dual-modality treatment of tumor hypoxia, showing enhanced antitumor effects (Huang et al., 2015; Sheng et al., 2017). Here, the functionalization of OLNBs surface with SPIONs to obtain MOLNBs is reported. This new specific nanoscaled carrier demonstrated to be effective as locally hyperthermic agent in *in vitro* studies.

Magnetic oxygen-loaded nanobubbles have a well-defined hollow structure, whose shell is densely decorated by SPIONs, as shown by TEM analysis. The electrostatic binding between the SPION positive charge and the negative surface charge of dextran sulfate–shelled NBs is strong enough to produce a stable nanostructure.

The preliminary hyperthermic investigations of this work confirmed that MOLNBs might be a promising new theranostic MFH agent, presenting the additional advantages of delivering oxygen to cancer cells and allowing their imaging detection.

Interestingly, under RF magnetic field, MOLNBs manufactured with an SPION concentration of 2 mg/mL produced a temperature increase as large as 5°C at field amplitude of 250 Oe and 7°C at 300 Oe, after 10 minutes of treatment. The calculated SAR values of MOLNBs demonstrated a good heating efficiency and were comparable with those recently obtained by Umut et al. (2019), Arosio et al. (2019), and Sudame et al. (2019).

The magnetic and hyperthermic properties can be possibly related to the small dimensions of the SPIONs and the average number of SPIONs that decorated the OLNB shell. As a matter of fact, preliminary investigations by Albertini et al. showed that the thermic effect can be related to the presence of SPION clusters, reaching higher temperatures in the presence of increased SPION agglomeration (personal communication). Therefore, based on these results, MOLNBs could represent an innovative multitheranostic approach for hyperthermic treatment.

In addition, a significant and sustained *in vitro* oxygen delivery from MOLNBs was detected *in vitro*. Importantly, the presence of SPIONs on NB shell did not affect the oxygen release kinetics in comparison with OLNBs.

Oxygen release from NBs is due to passive gas diffusion across the shell and is mainly controlled by the difference in the gas concentration between the core and the external environment. Interestingly, MOLNB core, being composed by PFP in which oxygen has a very high solubility, can act as a gas reservoir from which oxygen can be slowly released with kinetics depending on its partial pressure, according to Henry law. Moreover, it is necessary to take into account for the oxygen release kinetics the high internal NB pressure that is inversely correlated with the bubble radius, according to the Laplace law.

Furthermore, the oxygen delivery from MOLNBs could be tuned for specific hyperthermic treatment by modulating the nanostructure of the nanocarriers, i.e., shell thickness and particle diameter.

According to **Figure 8**, the temperature increase in the hyperthermic range is responsible for a sustained enhancement of oxygen release over time, which determines an oxygen concentration increase of about 0.5 mg/L in the external aqueous solution. Such effect is possibly related to modifications in shell permeability, associated to changes of the MOLNB nanostructure. Indeed, phospholipids underwent sol-gel-sol transition in function of the temperature. The long-lasting oxygen release over time might be expected in heated biological tissues and would contribute to their oxygenation in addition to the physiological mechanisms related to the heat-induced hyperemia.

Unfortunately, we were unable to directly record the oxygen delivery in the presence of a magnetic field. Indeed, direct measurements were prevented when MOLNBs were in such conditions, due to the metallic composition of the oximetry probe. In our experimental setup, the oxygen concentration was measured just after the withdrawal of the sample from the magnetic field. We observed that the sustained *in vitro* oxygen release profile was maintained also after irradiation. This might indicate the integrity of the NB structure. The occurrence of mechanical modifications of the MOLNB shell following the interaction with RF magnetic field could be excluded, because no massive oxygen delivery after the hyperthermic session was observed.

The theranostic nature of the MOLNBs was confirmed by preliminary US imaging, showing the system echogenicity. For both OLNBs and MOLNBs, the acoustic contrast resulting from B-mode imaging increased when the temperature increased from room to body temperature. The echogenicity of NB is related to droplet-to-bubble phase transition.

Indeed, PFP-loaded NBs present the ability to be activated by US, by means of a phenomenon called ADV. Acoustic droplet vaporization allows nanodroplets to be converted into bubbles following a US pulse, inducing the liquid-to-gas transition within the bubble core (Kripfgans et al., 2004). However, the MOLNBs revealed an enhanced acoustic contrast with respect to OLNBs, probably depending on the difference in compressibility and density with respect to water.

Finally, *in vitro* biological studies confirmed that MOLNBs can be easily internalized by tumor cells, and no cytotoxic effects were observed.

## Conclusions

We prepared and characterized superparamagnetic theranostic OLNBs with echogenicity, heating potential, and internalization capability in a cancer cellular model. Magnetic oxygen-loaded nanobubbles may be a new interesting nanotool for multifaced tumor treatment, both inducing HT upon RF magnetic field exposure and releasing oxygen as radiosensitizer for RT. This nanoformulation might provide a new approach to improve the therapeutic cancer outcomes, allowing to locally kill cancer cells by heating the tumor tissues at hyperthermic temperatures.

## Data availabilty

All data sets generated for there study are included in the manuscript and the supplementary files.

## Author Contributions

MA and SA formulated, prepared, and *in vitro* characterized the NB nanoformulations; LN performed TEM analyses; SZ performed US imaging characterization; RCi and FA carried out hyperthermic and magnetic measurements; CG and RCa designed the experiments and gave the intellectual rationale to the work.

## Funding

The present work was supported by funds from the University of Turin (ex 60% to RCa and Finanziamento Ricerca Locale 2018 - Linea B to MA).

## Conflict of Interest Statement

The authors declare that the research was conducted in the absence of any commercial or financial relationships that could be construed as a potential conflict of interest.
